# Exposure to Secondhand Smoke in Homes and Vehicles Among US Youths, United States, 2011–2019

**DOI:** 10.5888/pcd17.200107

**Published:** 2020-09-10

**Authors:** Kimp Walton, Andrea S. Gentzke, Rebecca Murphy-Hoefer, Brandon Kenemer, Linda J. Neff

**Affiliations:** 1 Office on Smoking and Health, National Center for Chronic Disease Prevention and Health Promotion, Centers for Disease Control and Prevention, Atlanta, Georgia

## Abstract

In this study, we report the prevalence of self-reported secondhand smoke (SHS) exposure in homes and vehicles among US middle and high school students in 2019 and changes in SHS exposure over time. Data were from 7 years of the National Youth Tobacco Survey (NYTS; 2011, 2013, and 2015–2019). In 2019, 25.3% (an estimated 6.7 million) of students reported home SHS exposure and 23.3% (6.1 million) reported vehicle SHS exposure. Home and vehicle SHS exposure significantly declined during 2011 through 2018, except for home exposure among non-Hispanic black students. Implementation of smoke-free policies in public and private settings can reduce SHS exposure.

**Summary**

**What is already known on this topic?**

Secondhand smoke (SHS) harms children and adults, and the only way to fully protect nonsmokers is to eliminate smoking in indoor environments.

**What is added by this report?**

In 2019, 25.3% (an estimated 6.7 million) of US middle and high school students reported home SHS exposure, and 23.3% (6.1 million) reported vehicle SHS exposure. SHS exposure in homes and vehicles significantly declined during 2011 through 2018, except for SHS exposure in homes among non-Hispanic black students.

**What are the implications for public health practice?**

Implementation of smoke-free policies in both public and private settings, including homes and vehicles, can help reduce SHS exposure, particularly among youths.

## Objective

The adverse health effects of secondhand smoke (SHS) exposure from combustible tobacco products are well established (1); there is no risk-free level of SHS exposure (2). Smoke-free policies can reduce SHS exposure and prevent tobacco use initiation and promote cessation of tobacco use. Although progress has been made in enacting comprehensive smoke-free indoor air laws in public settings, private settings such as homes and vehicles remain major sources of exposure for some populations, including youths. In this article, we report the prevalence of self-reported SHS exposure in homes and vehicles among US youths in 2019 and changes in SHS exposure during 2011 through 2018.

## Methods

Data were from the National Youth Tobacco Survey (NYTS), a cross-sectional, self-administered survey of US middle (grades 6–8) and high school (grades 9–12) students attending public and private schools. NYTS applies a stratified, 3-stage cluster sample design to produce a nationally representative sample of this population. Seven years of NYTS data (2011, 2013, and 2015–2019) were used. Because of a change in the mode of survey administration in 2019 (3), we were unable to compare 2019 results with previous years. Sample sizes ranged from 17,711 in 2015 to 20,675 in 2016, and response rates ranged from 63.4% in 2015 to 72.7% in 2011. SHS exposure-related outcome data were not available in 2012 and 2014.

Home and vehicle SHS exposures were determined from the questions “During the past 7 days, on how many days did someone smoke tobacco products in your home while you were there?”, “During the past 7 days, on how many days did you ride in a vehicle where someone was smoking a tobacco product?” (2011, 2013, 2015), and “During the past 7 days, on how many days did you ride in a vehicle when someone was smoking a tobacco product?” (2016–2019). For all outcomes, a response other than “0 days” was classified as being exposed.

In 2019, prevalence estimates and 95% confidence intervals were calculated separately for home and vehicle exposures among analytic subpopulations of nonusers of tobacco products, noncombustible tobacco product only users, and combustible tobacco product users. Combustible tobacco product users were either those who used combustible products only or both combustible and noncombustible products. SHS exposure was reported overall and by covariates (sex, school level, and race/ethnicity). We used χ^2^ tests to examine differences in SHS exposure prevalence by covariate levels in 2019. Changes in SHS exposure prevalence were examined between 2011 and 2018 for each covariate category. All analyses were conducted on weighted data by using SAS-callable SUDAAN version 11.0.3 (RTI International). Significance was set for all analyses at *P* < .05.

## Results

In 2019, 25.3% (an estimated 6.7 million) of middle and high school students reported home SHS exposure, and 23.3% (6.1 million) reported vehicle SHS exposure (Table). A significant difference in home SHS exposure was observed by race/ethnicity; non-Hispanic black (28.4%; 980,000) and non-Hispanic white (27.4%; 4 million) students both had a higher prevalence than Hispanic (20.0%; 1.3 million) and non-Hispanic other (20.2%; 290,000) students (*P* < .001).

**Table:** Prevalence of Self-Reported Exposure to Secondhand Tobacco Smoke in Homes and Vehicles During the Past 7 Days Among US Middle and High School Students, Overall and by Selected Characteristics, National Youth Tobacco Survey, 2019

Characteristic	Overall, % (95% CI)	Current Combustible Tobacco Use^a^, % (95% CI)	Current Noncombustible Tobacco Use Only^b^, % (95% CI)	No Current Tobacco Use^c^, % (95% CI)
**SHS Exposure in Home^d^**				
**Overall**	25.3 (23.4–27.3)	52.5 (48.7–56.2)	31.7 (28.8–34.8)	20.8 (19.1–22.6)
**School level**				
Middle school	25.9 (23.6–28.3)	62.2 (58.3–66.0)^e^	38.2 (32.3–44.5)^e^	22.6 (20.5–24.8)^e^
High school	24.9 (22.6–27.4)	49.5 (44.9–54.0)^e^	29.9 (26.5–33.5)^e^	19.1 (17.1–21.3)^e^
**Sex**				
Male	24.9 (22.9–27.1)	50.0 (44.7–55.4)	31.5 (28.2–35.1)	20.4 (18.5–22.5)
Female	25.7 (23.7–27.8)	55.8 (51.2–60.2)	32.0 (28.4–35.7)	21.3 (19.5–23.2)
**Race/ethnicity**				
Non-Hispanic white	27.4 (24.8–30.1)^e^	55.3 (50.2–60.3)^e^	33.6 (29.8–37.6)	22.7 (20.4–25.1)^e^
Non-Hispanic black	28.4 (24.6–32.6)^e^	52.2 (45.5–58.9)^e^	33.3 (24.9–43.0)	24.0 (20.3–28.2)^e^
Hispanic	20.0 (18.4–21.8)^e^	47.7 (42.8–52.8)^e^	27.1 (22.7–32.0)	15.5 (13.9–17.3)^e^
Non-Hispanic other	20.2 (16.5–24.4)^e^	37.6 (23.8–53.8)^e^	30.2 (19.9–43.0)	17.9 (14.4–21.9)^e^
**SHS Exposure in Vehicle^f^**				
**Overall**	23.3 (21.4–25.4)	56.3 (52.6–60.0)	34.7 (31.9–37.6)	17.4 (15.7–19.3)
**School level**				
Middle school	21.4 (19.0–24.0)^e^	58.0 (51.9–63.8)	33.0 (27.3–39.2)	18.3 (16.2–20.6)
High school	24.9 (22.6–27.3)^e^	56.0 (51.7–60.3)	35.2 (32.0–38.6)	16.7 (14.7–18.8)
**Sex**				
Male	22.7 (20.6–25.0)	55.0 (50.8–59.2)	34.8 (31.4–38.3)	16.5 (14.5–18.6)^e^
Female	24.0 (21.9–26.2)	58.0 (53.2–62.6)	34.7 (31.3–38.4)	18.5 (16.5–20.5)^e^
**Race/ethnicity**				
Non-Hispanic white	26.1 (23.5–28.9)^e^	62.4 (57.8–66.8)^e^	37.7 (34.3–41.1)^e^	19.4 (17.1–21.9)^e^
Non-Hispanic black	26.4 (23.4–29.7)^e^	50.6 (42.5–58.6)^e^	41.7 (32.9–51.1)^e^	21.2 (18.4–24.4)^e^
Hispanic	17.6 (15.9–19.3)^e^	48.9 (43.4–54.4)^e^	25.9 (21.9–30.4)^e^	12.6 (11.0–14.3)^e^
Non-Hispanic other	14.0 (11.2–17.4)^e^	31.3 (20.2–45.2)^e^	26.9 (17.5–38.8)^e^	11.6 (9.0–14.7)^e^

Abbreviations: CI, confidence interval; SHS, secondhand smoke.

a Combustible tobacco products are cigarettes; cigars, cigarillos, little cigars; pipe filled with tobacco; bidis; and hookah or waterpipe. Current users reported use of ≥1 of these products on ≥1 days of the past 30 days.

b Noncombustible tobacco products are e-cigarettes; chewing tobacco, snuff, or dip; snus; or dissolvable tobacco. Current users reported exclusive use of ≥1 of these products on ≥1 days of the past 30 days.

c Noncurrent tobacco product users reported use of combustible and noncombustible tobacco products on 0 days of the past 30 days.

d A response from 1 to 7 to the question “During the past 7 days, on how many days did someone smoke tobacco products in your home while you were there?”

e Significant difference in estimates for characteristic categories based on χ^2^ test (*P* < .05).

f A response from 1 to 7 to the question “During the past 7 days, on how many days did you ride in a vehicle when someone was smoking a tobacco product?”

For vehicles, a significant difference was observed by school level (high school: 24.9%, 3.6 million; vs middle school: 21.4%, 2.5 million; *P* = .01). Non-Hispanic black (26.4%; 880,000) and non-Hispanic white (26.1%; 3.8 million) students had higher prevalences of SHS exposure in vehicles than Hispanic (17.6%; 1.1 million) and non-Hispanic other (14.0%; 200,000) students (*P* < .001). SHS exposure in homes and vehicles was highest among students currently using combustible tobacco products (homes: 52.5%; vehicles: 56.3%), followed by noncombustible tobacco product only users (homes: 31.7%; vehicles: 34.7%) and nonusers of tobacco products (homes: 20.8%; vehicles: 17.4%) (*P* < .001) (Table).

Overall, home SHS exposures declined from 26.8% in 2011 to 20.9% in 2018 (*P* < .001); vehicle SHS exposures declined from 30.2% in 2011 to 19.8% in 2018 (*P* < .001) (Figure). For all characteristics, SHS exposure in homes and vehicles significantly declined during 2011 through 2018, except for SHS exposure in homes among non-Hispanic black students, in which it did not change.

**Figure:**
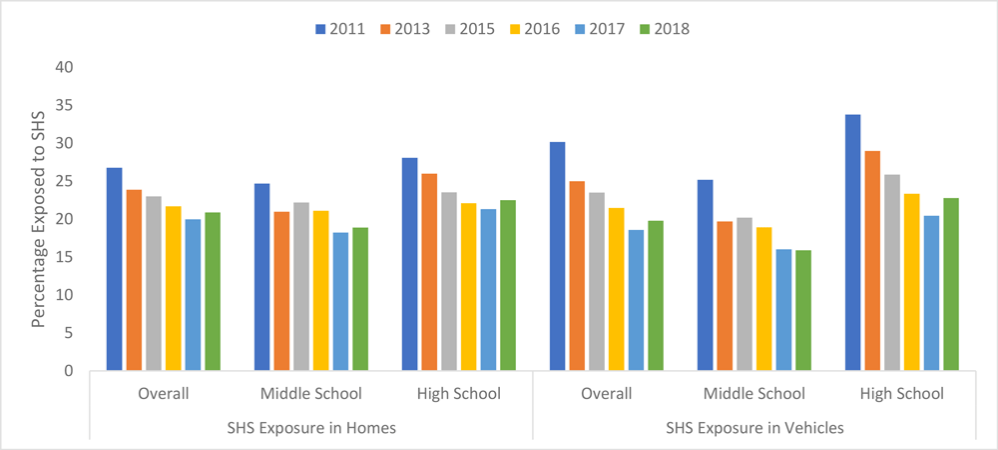
Prevalence of self-reported exposure to secondhand smoke (SHS) in homes and vehicles during the past 7 days among US middle and high school students, overall and by school level, National Youth Tobacco Survey, 2011–2018.

**Table:** 

Year	SHS Exposure in Homes			SHS Exposure in Vehicles		
	Overall	Middle School	High School	Overall	Middle School	High School
2011	26.8	24.7	28.1	30.2	25.2	33.8
2013	23.9	21.0	26.0	25.0	19.7	29.0
2015	23.0	22.2	23.6	23.5	20.2	25.9
2016	21.7	21.1	22.1	21.5	18.9	23.4
2017	20.0	18.2	21.3	18.6	16.0	20.5
2018	20.9	18.9	22.5	19.8	15.9	22.8

## Discussion

In 2019, approximately one-quarter of US middle and high school students reported SHS exposure in homes (25.3%) and vehicles (23.3%). Eliminating smoking in indoor spaces is the only way to fully protect nonsmokers from SHS exposure in these environments. Additionally, smoke-free policies can reduce tobacco use initiation, promote tobacco use cessation, and influence social norms by reducing the social acceptability of combustible tobacco product use (4).

During the past 2 decades, progress has been made toward reducing SHS exposures in the United States (5). To date, 27 states and more than 1,000 municipalities have implemented comprehensive smoke-free laws that prohibit smoking in indoor public places, including workplaces, restaurants, and bars (6,7). These policies can also promote increased adoption of voluntary smoke-free rules inside homes and private vehicles (8). With the enactment of smoke-free laws, it also is important to address and monitor the impact on SHS exposures in these private settings. For example, a 2018 rule by the US Department of Housing and Urban Development made all public housing smoke-free (9), and 9 states and 1 US territory have passed laws prohibiting smoking in vehicles occupied by children (10).

Although the prevalence of voluntary smoke-free home (83.7%) and vehicle (78.1%) rules has increased over time (11), these private settings remain major sources of SHS exposure for many people, including youths (2). Similar to findings in other studies, our results show disparities by race/ethnicity; non-Hispanic white and black students had a higher prevalence of SHS exposure in the home compared with Hispanic students and students of other races (5,12). Furthermore, high school students reported a higher prevalence of SHS exposure in vehicles than did middle school students. These differences could be reduced with the implementation of smoke-free policies in additional environments.

This report is subject to limitations. First, the survey is limited to middle and high school students, so results aren’t generalizable to all US youths. Second, SHS exposures were self-reported and not verified with nicotine biomarkers. Additionally, response rates ranged from 63.4% to 72.7%; thus, results may be subject to nonresponse bias. Finally, because of the change in the mode of survey administration in 2019 (from a paper-and-pencil instrument to a digital tablet), we were not able to compare 2019 results to 2011 results.

In 2019 approximately one-quarter of US middle and high school students reported SHS exposure in homes and vehicles. Although SHS exposures have declined, more than 6 million young people remain exposed to SHS in these private settings. Implementation of smoke-free policies in both public and private settings, including homes and vehicles, can help reduce SHS exposure, particularly among youths.
